# ‘No Dumping!’ Threshold Work in Voluntary Sector Mental Healthcare in Inner‐City London

**DOI:** 10.1111/1467-9566.70264

**Published:** 2026-09-24

**Authors:** Natassia Brenman

**Affiliations:** ^1^ Nuffield Department of Primary Care Health Sciences University of Oxford Oxford UK

## Abstract

This paper examines the sociomaterial dynamics of mental healthcare by following metaphors circulating among voluntary sector providers that they were becoming ‘dumping grounds’ within a system in crisis. In the United Kingdom, the voluntary sector has long been called upon to deliver flexible, community‐based care that reduces pressure on primary and secondary services. Under chronic conditions of austerity, however, providers have been stretched beyond their capacity. Drawing on ethnographic research across three voluntary sector mental health sites in inner‐city London, I analyse how providers made and maintained places of care while grappling with expanding and shifting demands. Taking a sociomaterial approach, I propose the analytic concept of ‘threshold work’ to complicate the dumping metaphor and to reconceptualise how material thresholds of place and immaterial thresholds of mental health need are co‐constituted with care practices. I identify three interrelated modes of threshold work through which providers manage the limits of inclusion, revealing how diagnostic boundaries of need and spatial arrangements of care move together, shaping the terms and conditions of care in the city.

## 1 Introduction and Background

‘No Dumping!’ says the makeshift sign, imploringly hand‐painted directly onto the wall of the cul‐de‐sac, where a voluntary sector counselling service is based in inner‐city London. The sign is much needed here, as the land is privately owned, meaning there are no rubbish collections. But, despite its size and hefty underline, the sign cannot do all the work: Full bin bags regularly appear outside the service, which staff normally pick up and take to the end of the street, past the threshold that the bin men will not cross. The abandoned cars that line the street press into the already narrow space outside the doors of the service, resting on soft tyres, which threaten to sink into the patchy tarmac below. I am joining the tail end of a big clean‐up of the area outside the service. The counselling staff and volunteers have decided to spend a few days making it look more welcoming for their clients: people struggling to cope with trauma or loss, and in particular, people who would not normally seek help through psychotherapy.

As they clear the last bits of stray litter and put the finishing touches to the newly painted sign, the staff worry about the shards of glass that shine in the gutter, and—a new development—the corner next to some loose breeze blocks, which Sian, the director of the service, says ‘someone’ has been using as a toilet. Shocked at her assumption that it was a human ‘someone’, I ask her why she does not think it was just a dog or a fox. She reminds me of a story I had heard before about two homeless people who used to sleep and hang around in the cul‐de‐sac. One of the administrative staff had described them in benign—although rather euphemistic—terms, saying several times that they ‘weren't lovable’, gently explaining how they had unhygienic habits of ‘making a mess’, peeing outside or sleeping with their dog lying between them. Others were more hostile, like the volunteer counsellor who wanted me to know that he did not believe they were homeless at all, calling them ‘street drinkers’ instead. The worry was that they would scare vulnerable clients approaching the service for counselling. In the end, the police had been called to move the homeless people on. Now, Sian is concerned the excrement is a sign of their return. She tells me she is toying with the idea of erecting some gates down at the entrance to the cul‐de‐sac, where it meets the busy high street, to try to keep people who are not residents or clients from coming into the space. And so, it is not just ‘matter out of place’ that these mental health service providers are concerned about; it is also *people* out of place (Douglas 1966; Ahmed 2000).

This particular scene happened in isolation to the day‐to‐day problems of accessing and providing care that I was charting in my fieldwork, based at a consortium of three voluntary sector mental health services in inner‐city London between 2016 and 2018. But it was a moment in which I saw an uncomfortably familiar metaphor materialise, quite literally writ large on the walls of this place of care. ‘No dumping!’ said the sign, whereas just metres away, inside the doors of the service, I had heard talk of voluntary organisations being at risk of becoming ‘dumping grounds’ and referrals from mainstream services described as ‘dumping’. Inside the service, some weeks earlier: Sara is standing in the doorway of the office before her client arrives for their therapy session. She is telling me how people who get referred by mainstream services always seem to be more likely to miss appointments than those who self‐refer. But there is something else she wants to get off her chest. ‘These people’, she says, ‘they're the ones that the other services can't deal with, or they don't know what to do with… They're not meant for here. This one woman I see now, she's totally psychotic, it's really tricky…’ She goes on, explaining that with so many people being referred from crisis houses and other oversubscribed services, it is hard to tell which cases are ‘genuine’. By this, she means those who need weekly counselling rather than support in an acute mental health crisis and/or severe and enduring mental health issues. She turns to leave finally but lingers, saying after a pause, ‘You know, this place is becoming a dumping ground, I'm telling you. It's a dumping ground for these other services…’

Of all the spatial metaphors that arose in the day‐to‐day talk in these services (bridges, sanctuaries, holding spaces and so on), the dumping ground carried with it the most troubled meanings and associations. The metaphor spoke to the widely held concern that these services were becoming increasingly and unfairly overburdened in a climate of overcrowded and underfunded mental healthcare, despite the economic logics driving mental health policy at the time (Pickersgill 2019). This sits within a broader context of what anthropologist Fiona Wright has described as "the UK's chronically ‘crisis‐stricken’ mental health‐care system" (Wright 2022, 1), spanning from the shift to ‘community care’ in the 1980s to the huge mental health budget cuts of the austerity measures brought in in response to the financial crisis of 2008 (Wright 2022). In the decade leading up to my fieldwork between 2016 and 2018, the voluntary sector had been positioned as ‘a shock absorber in a time of need’ (Foster 2015, 118). Although overall state spending on health and mental health services has risen significantly since then, the voluntary sector has continued to absorb pressures from mainstream services (Sambrook et al. 2025), and problems around access and the ‘conditionality’ of mental healthcare provision have persisted (Kiely 2024). Voluntary and community sector services have been mandated to address only less severe need (often described as ‘common mental disorders’) but are often pressured to take on much more complexity and severity, as illustrated in the ethnographic vignette above.

When severely unwell people end up in community counselling services, mental health needs can be seen to be ‘spilling over’ both diagnostic eligibility thresholds *and* the limits of provision (capacity, space, staffing and so on). The already contested metaphor of the postpandemic ‘tsunami’ of mental health problems (Fitzgerald et al. 2020; Shevlin et al. 2021) is a powerful image, which conjures up impressions of overflow and the danger of boundaries being breached. If certain places of care continue to be considered at risk of becoming ‘dumping grounds’, there is an urgent need to address the sociomaterial and ethical dynamics at play at these breach‐able thresholds. These stakes highlight how carefully these metaphors of dumps and dumping must be handled, not least because of their strong connotations with overflow, dirt and carelessness—highly charged terms in the world of mental health service provision. They carry the risk of simplifying and stigmatising the complex social, clinical and spatial problems of providing and receiving care under conditions of austerity. And yet, I argue that observing their use in practice and ‘in place’ (N. Brenman 2023) draws out important patterns within the labour of inclusion and exclusion: the messy work of care at the edges (Traill et al. 2024).

In this paper, I explore how metaphors and materialities of ‘dumping’ might provide an opportunity to better understand the more complex *threshold work* in spaces of mental healthcare. Occupying a theoretical intersection between medical sociology, anthropology and feminist science and technology studies (STS), I propose new ways to think about matter, and people, ‘out of place’. I take the imperative ‘no dumping!’ as an empirical starting point for understanding how voluntary sector care providers make and maintain spaces of care on the peripheries of overburdened mainstream service provision. Specifically, I chart how the problem of access is managed by tending to thresholds of place, and of need, in everyday practices of tackling exclusion in the voluntary sector. I also take ‘no dumping!’ as a provocation to move away from the abstract metaphor of ‘dumping’ and how it can manifest within the dynamics of mental health service provision. I propose the analytic concept of ‘threshold work’ to complicate this metaphor and to reconceptualise how material thresholds of place and immaterial thresholds of mental health need are co‐constituted with care practices. This mode of analysis reveals how diagnostic boundaries of need and spatial arrangements of care move together, shaping the terms and conditions of care in the city.

## 2 ‘Placing’ Psychiatric Subjects

There is now a rich body of literature in the sociology of health and illness, which conceptualises place and materiality as co‐constituent with care practices (Oudshoorn 2011; Bell 2018; Montgomery et al. 2024). This focus on space and place draws and builds on human geography, including Hester Parr's seminal work on ‘micro‐geographies’ of inclusion and exclusion in a de‐institutionalised landscape of mental healthcare (Parr 1997, 2000). There has been a recent emphasis on the shifting, flexible nature of places in a digital age, described as ‘post‐place care’ (Ivanova 2020), though recognition of the fluid and contingent nature of places is not bound to digital care. In their paper on ‘placing work’ in end‐of‐life care, Driessen et al. (2021) have pointed to the dynamic relation between a patient and their location as they approach the end of their life. Speaking to themes of ‘matter’—and people—‘out of place’, Driessen and colleagues point to the ways in which Douglas’ (1966) classic work on the link between the material and the symbolic can paradoxically reinforce ideas about the fixed nature of material things and places. This is something that has been challenged by social theory concerned with sociomaterial practices of ‘mattering’ the world (Barad 2003), where the material world is co‐constituted with immaterial systems of order (such as social, moral or even clinical categories). Extending these conversations, I turn to the co‐constituent nature of the *limits* of place and care: the nexus between material boundaries of place, upper and lower thresholds of need, and care practices.

In the sociology and anthropology of mental health, much attention has been paid to the shape‐shifting and socially contingent nature of diagnostic boundaries in psychiatry (Busfield 2000). More recently, psychological models of mental health have been rendered in relation to a similarly flexible typology. They focus on ‘common mental disorders’, defined as low‐level anxiety and depression, with newly defined upper and lower thresholds of ‘meetable’ psychological need (Pickersgill 2019). Ethnographic scholarship has developed these ideas around flexible categories of need in relation to eligible subject positions. For example, Fassin (2012) conceptualises the fashioning of ‘kinds of people’ (or ‘subjectivations’) by humanitarian health practitioners who need systems to identify and triage individuals with complex mental health needs. Kienzler (2012) uses this idea to understand how practitioners in resource‐scarce settings ‘make people fit’ ready‐made diagnostic categories such as PTSD as an ethical practice of inclusion. As such, the shaping of diagnostic boundaries and categories of mental health need has largely been understood in medical sociology and anthropology in relation to subject positions, rather than the spaces or places of mental healthcare. This has remained largely separate from the rich work in human geography around the organisation and practice of mental healthcare (Parr 2000; Milligan and Conradson 2006; Kiely 2021).

In sum, there has been a strong focus on the co‐constituent nature of place and care in medical sociology, and a longstanding interest in the flexibility of diagnostic boundaries in relation to available care and eligible subject positions, particularly in the sociology and anthropology of mental healthcare. There has been less focus on the nexus between the material boundaries of place, thresholds of mental health need and care practices, which the talk of the ‘dumping ground’ in my ethnographic work pushed me to attend to. There often remains an underlying assumption that places of care and their material boundaries are fixed and exist outside of these processes of categorising people and needs in mental health. Notable exceptions come from empirical work informed by assemblage theory and sociomateriality. For example, McGrath and Reavey (2016) have linked the dissipation of mental health services into community spaces to the modes of assessment and service delivery, describing the ‘space time’ of both care and service user distress. In addition, Bister et al.’s concept of niching in infrastructures of mental healthcare helps to understand ‘patients, their (mental) needs, the psychiatric care system and related housing arrangements as interacting entities’ (Bister et al. 2016, 192). This pushes us to map the sociomaterial conditions from which practices of in/exclusion in mental health emerge. In this paper (and special issue), this relates to conditions of scarcity and precarity as they are embedded in urban assemblages (Bieler and Klausner 2019), sociomaterial infrastructures (Berlant 2015), and in the relations between urban citizens and the spaces they inhabit (Rose and Fitzgerald 2022).

In my sociomaterial approach to understanding the ‘placing’ of psychiatric subjects and providing access to care, I ask: What is the relationship between thresholds of place, and need, and care practices that co‐constitute them? As the boundaries of diagnostic categories shift in tandem with changing forms of sociality and subject positions, do places of care simply exist as containers waiting to be filled? The dumping ground metaphor makes clear that places are far from neutral containers, but how do we take seriously the stark power of this metaphor, while also challenging the static binaries it suggests, about inside and outside, and value and waste? The analysis that follows suggests that thresholds of place and thresholds of need are both fragile and contingent on sociomaterial practices of inclusion and exclusion: practices that determine *who belongs where* in mental healthcare (N. F. Brenman 2019, 2020). Specifically, I argue that the analytic concept of threshold work provides insight into the ongoing production of both material thresholds between inside and outside (the politics of placemaking) and the immaterial thresholds of mental health need and eligibility.

## 3 Field Sites and Ethnographic Methodology

Over a period of 18 months, I worked closely with three organisations providing psychotherapy services within a diverse, inner‐city context in London. In the years leading up to my fieldwork, their relationship to the mainstream became particularly complex with the introduction of the ‘Any Qualified Provider’ policy of the Health and Social Care Act (2012). This made explicit its reliance on voluntary services to provide care to ‘underserved and specialised groups’ who find it harder to access services via mainstream clinical routes in the National Health Service (NHS). Care became organised by NHS clinical commissioning groups, tasked with purchasing the provision of healthcare services within a government‐regulated market (Wright 2022). Two of the three organisations were operating under such contracts, and all three worked as a consortium to cover a cluster of inner‐city London boroughs with high levels of diverse mental health need—a reflection of the extremely wide gaps between rich and poor in a ‘superdiverse’ area (Vertovec 2007), which has seen many waves of migration and demographic change.

All three services had been established and were run largely by the communities they considered to be their ‘client group’—communities that would often be referred to in the now contested language of the ‘hard to reach’ (see Kasstan 2020). One centre, which I call ‘Culture in Mind’, was run for and by people from ethnic, linguistic and cultural minorities, specialising in (but not restricted to) working with migrants and refugees. A second provided psychotherapy exclusively for women. The ‘Pankhurst Women's Centre’ (also a pseudonym) was a feminist project that reached out to socially marginalised women, focusing—at the time of my research—on survivors of gender‐based violence, trafficking and forced migration. The third centre (introduced in the opening of this paper) provided support to anyone suffering from experiences of trauma or loss who would not normally seek help through psychotherapy. I call this the ‘Stepping Stones service’. As a consortium, these services aimed to make talking therapy more accessible to people who are typically absent from both public (state‐provided) and private (paid‐for) psychotherapy.

As the study took shape as an ‘ethnography of access’ (N. F. Brenman 2019), I sought to disrupt the politically neutral narratives of ‘barriers and facilitators’ that often frame problems of access to care in health services research. Instead, I situated these problems in very specific social, economic and material arrangements, focusing on threshold spaces in the institutional and material landscape of care provision. I have described this methodology elsewhere in terms of ‘writing through mess’—both the material untidiness of inner‐city spaces of care and the messy politics of inclusion (N. Brenman 2023). Taking seriously the role of doors as sites of moral negotiation in the architecture of psychotherapeutic settings (d’Hoop 2022), these threshold spaces included entrances, front desks and doors to the waiting rooms and clinical spaces, as well as other points of access such as referrals and waiting lists. Participant observation involved shadowing therapists, clinical managers, community development therapists and link workers; observing clinical and organisational meetings; and participating in and volunteering at events such as conferences, presentations, training sessions and the induction of new trainee therapists. I also draw on interviews I carried out with 14 therapists and staff members, most of whom I interviewed two or more times across the fieldwork period. These were recorded, transcribed and analysed based on emerging themes, alongside ethnographic field notes. Ethical approval for this study was granted by the London School of Hygiene & Tropical Medicine Research Ethics Committee. Written informed consent was obtained from all participants, and ongoing verbal consent was sought during participant observation in accordance with ethnographic research practice. To protect the identities of my interlocutors, I have replaced all names of people and places with pseudonyms and changed some details of the organisations.

My analysis of these data focused on material and discursive ‘threshold work’ in these settings: the labour of managing access via clinical thresholds of eligibility and the physical thresholds of the clinic. The concept of threshold work was generated in an iterative process of analysing the ethnographic material in relation to theory in an abductive manner (Cubellis et al. 2021), for example, exploring the notion of ‘boundary work’ in relation to symbolic categories (Gieryn 1983) and adapting it to relate to encounters at the material boundaries of care settings in the urban environment. I was also informed by the feminist STS focus on the (often hidden) maintenance and repair work (De Laet and Mol 2000; Mattern 2018), which was relevant to the care practices I was observing in a system that is routinely described as ‘broken’. Finally, I critically analysed the metaphor of dumping that I encountered in the field using Cowan and Rault's approach to ‘grappling with the material histories and consequences of metaphors’ (Cowan and Rault 2022, 9). Their orientation towards the knowledges and material practices from which a metaphor might gain purchase enabled me to interrogate and complicate the metaphor of dumping that I encountered in the field. This generated three distinct but interrelated modes of threshold work: ‘making space’, ‘allowing overspill’ and ‘tidying up’. Each articulates a relational practice of in/exclusion: how mental healthcare providers grapple with and manage the limits of inclusion to care in the city.

## 4 Making Space

All three of the voluntary sector psychotherapy clinics I worked with explicitly sought to ‘make space’ for people who do not easily fit into mainstream mental health settings. However, in one of these sites—the Pankhurst Women's Centre—this process of making space was particularly bound up with sociomaterial practices of flexibly creating, adjusting and shifting physical places of care. Here, these practices were salient, firstly, because of the high value and stakes of providing a safe place for vulnerable women, and secondly, because of the ongoing challenges they faced in delivering this. The organisation had been set up in the 1970s during the women's liberation movement, starting out in the basement of one of the founder's houses. This origin story was often narrated by the women who worked there, reflecting ongoing values of self‐sufficiency, ownership of mental healthcare for and by women, and alternative approaches to mainstream psychotherapy.

Several decades on, they had long since moved out of the basement into a complex of sturdy, nineteenth‐century red brick buildings accommodating several community‐based health and welfare charities. They continued to provide courses of one‐to‐one and group therapy in a space that deliberately contrasted with more medicalised psychiatric settings or mainstream psychological services. I spoke to one young woman who was in weekly one‐to‐one therapy at the centre, having previously spent time in a psychiatric ward when she was diagnosed with postnatal depression. She stressed the significance of these material differences, describing the small scale of the centre, the light and the pastel‐coloured art on the walls in contrast to the medical spaces and temporary accommodation she had found herself in when she was in crisis. The organisational and clinical staff felt protective of the space they had created or maintained, which both staff and clients would often refer to as a ‘sanctuary’.

The practicalities of maintaining this space and making it accessible for those who needed it were a constant struggle, which almost always came down to financial resources. They were the only site I worked with who did not hold a contract to deliver core mental health services under the national health policies described above, meaning they relied on short‐term, ‘soft’ charity funding which was never guaranteed. About 6 years before I started my fieldwork there, the organisation found themselves unable to afford the rent for all their therapy rooms. They sold off about two thirds of their floor space to the (then new) national programme for Improving Access to Psychological Therapies, commonly known as IAPT (Pickersgill 2019). This physical proximity to mainstream clinical psychology services did not sit well with the therapists and volunteer staff at the Pankhurst Centre, who did not identify with the service they now shared the building with. They felt the short‐term cognitive behavioural therapy delivered down the corridor from them was driven by economic efficiency, too standardised, and lacking in the specificity of the feminist psychodynamic methods they used. They felt protective of their sanctuary and worked hard to separate their service from the cool, municipal healthcare setting on the outside of the door. A new door in the central corridor sealed them off from each other completely. I learnt that service users and staff valued the interiority of the clinic space, with many considering it to be a site of protection from a less welcoming, sometimes harmful landscape of mainstream mental healthcare. As such, the spatial division between the services was a manifestation of both their shrinking capacity and their relations with the broader mental healthcare system.

In an echo of the concerns around voluntary sector services being ‘dumped on’ by other mainstream services, one of the senior clinicians who managed the allocation of new clients described their experience of being ‘bombarded by referrals from down the corridor’. These referrals were coming from the NHS IAPT service, which was not allowed to take on cases beyond certain thresholds of severity and complexity. The complaint was voiced during a clinical meeting in which they reviewed new referrals on the waiting list against a large A3 sheet summarising each funding stream, the specialism and client group that were eligible for it and their current capacity for new clients. It was an expression of resentment that they were struggling to keep up with the demand for specialised psychotherapeutic care, having been forced to downsize and reduce their capacity. However, for me, the more interesting response played out over a longer period and emerged as an alternative way to manage capacity and access under these circumstances. The response was being led by a woman I call Jehona—a community therapist and outreach worker who identified strongly with her own refugee status. Instead of working to protect the spatial and professional boundaries that gave shape to a fixed ‘sanctuary’, she actively worked to allow it to shape‐shift and move around. She would make space for the so‐called ‘hard to reach’ in her projects focusing on young mothers, and refugee and asylum‐seeking women. Taking her clinical practice to places that would be inaccessible to traditional psychotherapists, she ran taster sessions, psychoeducation and support groups in the corners of community centres, libraries, advice services and cultural centres in the wider landscape of the city.

In this way, Jehona successfully extended the centre's promise of sanctuary beyond its own thresholds, often in very deliberate ways, to include a changing client group under conditions of scarcity. This mode of threshold work emerged out of a need to maintain a commitment to reaching the most marginalised women in their community in the face of their shrinking capacity both in terms of physical space and their clinical capacity to take on a higher number of more complex cases. It opened up more capacity and fitted well with their ambition to provide care for increasingly marginalised groups, and yet it *also* destabilised the very notion of sanctuary and contradicted values around safe and protected spaces. These examples of threshold work animate how places of care are often made and remade in response to changing (sometimes conflicting) patterns of demand, values and capacity.

## 5 Allowing Overspill

In a clinical meeting in another centre, Culture in Mind, an ‘outsider’ has come to visit. The usual cosy circle in which new referrals and complex cases are discussed in depth by the senior therapists is interrupted by a PowerPoint presentation. We are all sitting a little straighter than usual. The ‘outsider’ is someone from the local IAPT team, come to tell us about how the mainstream *recovery system* works and how the centre can improve its recovery rates. The organisation must know this because (unlike the Women's Centre) they have been contracted and funded by the NHS, so are monitored using the same system as mainstream mental health services. This poses some serious problems, given that for the past 30 years they have been deliberately working *outside* this system, delivering mother‐tongue psychotherapy to people from ethnic, linguistic and cultural minorities. The problem being raised today is that they are nowhere near hitting the national target of at least 50% recovery rates, which in theory they need to be reaching if they want their NHS funding to continue. There are high hopes that the visitor will play the much‐needed role of mediator between this small service and the spectre of IAPT, with its stark measures of mental illness and recovery and its unreachable targets.

As the presentation rolls on, and the interjections come in thick and fast, I realise this debate about improving recovery rates is all about discerning which needs they are (and are not) responsible for trying to meet: what falls within their remit and what spills over the limits of their responsibility. This underpins fraught questions about ‘who belongs where’ across the mental health landscape. The therapists are being asked to adopt new gatekeeping practices, only taking on clients who fall into the ‘depression’ category of common mental disorders and Levels 2 and 3 on the mental health clustering scale for severity.^1^ The reward will be that their recovery rates will be better and their contract secure, but in order to get to this point, they will have to make sure that the clients they take on are—the visitor chooses his words carefully—‘IAPT friendly’. As the back and forth goes on, and senior therapists protest, stressing the consequences of ignoring the complex and sometimes severe needs potential clients come to them with: ‘they will have *nowhere* else to go!’ Towards the end of the meeting, the advice from our visitor becomes starker: ‘You really need to work hard at choosing people who fit into *the* “*IAPT box*.”’

The unfortunate choice of ‘the IAPT box’ metaphor only intensified the therapists' sense of being limited by the constraints of this new system. They were highly sceptical of ‘putting people in boxes’, and the whole value system and rationale of the intercultural therapy centre is based around being sensitive and responsive to *different kinds of* needs. Before long, a workaround emerged. I became aware of this workaround as I was shadowing K, the clinical manager, on a particularly busy day. She had agreed to let me join her as she took a moment to sit down and screen several new referrals that had come through—a process which is necessarily ad hoc, given that referrals must be looked at *before* they enter their system, reducing the risk of them being unnecessarily added to the (consistently overloaded) waiting list. I lean across the outspread files to watch her stripe each relevant piece of information on the referral notes with a highlighter, commenting under her breath as she goes. The information she is highlighting will help justify why these cases have been earmarked to be ‘IAPT‐Plus’: a strange category, which I had not heard before. The audible commentary and bits of highlighted text materialise as a fragmented list in my field notes:

An Iranian woman of 39: ‘Domestic Violence’, ‘Loss of husband after leaving eight months ago’, ‘Potentially a child bride when married’.

…

An asylum‐seeking Kurdish man: ‘recently migrated from Iran, to Iraq, to the UK’ ‘PTSD’ [she brackets this, saying she is not keen on the term, adding her own notes to the margin:] ‘Family shot in front of him,’ ‘asylum claim rejected’.

…

A British Caribbean man: ‘homeless’ ‘in and out of psychiatric care’, and ‘a previous client at the Centre’ [he called the service after his file was closed, saying ‘help me’, I am told]

As we take a last look over the final list of referrals, the highlighted text makes the case for these people's need to access care painfully clear. And yet it also seems to massively exceed the remit of a community service commissioned to provide talking therapy for low‐level ‘common mental disorders’. This, I find out, is what ‘IAPT‐Plus’ is for: a label that has recently been made available to services which are ‘similar’ to mainstream talking therapy services but which have been contracted to enable the inclusion of people facing different, more severe problems. It has been introduced amid the difficult, ongoing negotiations between local clinical commissioners and voluntary sector services about the parameters of ‘appropriate need’ associated inclusion and exclusion criteria for the service. In this centre, the parameters of need had to extend to people who had experienced adverse or traumatic life events, discrimination, difficult migration experiences, cultural dislocation and socioeconomic factors such as poor housing, unemployment and poverty. These experiences complicated and exacerbated so‐called ‘common mental disorders’, shaping mental ill‐health in ways that far exceeded the parameters of the IAPT box. The strangely permissive overspill category of IAPT‐Plus opened up more possibilities for legitimately taking on people whose needs could not be met under the standard IAPT framework. Allowing the need to spill out of the ‘IAPT box’ was what made this tactic work, but it was also what put the organisation in a precarious position: foregoing impressive recovery rates, stretching their own capacities and breaching carefully constructed thresholds of ‘appropriate need’.

## 6 Tidying Up

Sitting alongside and in tension with their longstanding ethos of keeping ‘open‐door policies’, the staff in all three services were having to think seriously about how to manage the increasing volume of people being referred to them and the severity of their problems. This came into sharp focus in a clinical meeting at the Stepping Stones service, where they had traditionally taken on ‘anyone’ in the community who had suffered trauma or loss in their lives. However, it was now becoming increasingly important to consider this suffering in relation to the clinical thresholds of severity and type described above. The meeting was taking place over strong breakfast tea and a table smattered with biscuit crumbs. The therapists and clinical director were talking about the range of different people who come into the service these days; Sara is uneasy about the kinds of problems she has been seeing recently. In addition to their experiences of trauma and loss, one client is very paranoid, and another has severe panic attacks that affect her attendance. As she trails off, Sian (her supervisor and the clinical director) interjects, wondering how the clinical assessments were allowing for this. Sara thinks, eyes raised to the ceiling. ‘Well, it was clear from her notes what the issues were, but we were never going to turn them away’. Ralph turns to me, knowing that this is something I am interested in, explaining gently that, ‘once someone has come through the door here, we hardly ever turn them away. So, in a way, the assessment almost isn't there to decide if they're appropriate’. Sian shakes her head ever so slightly, waiting for Ralph to finish. Then she changes tack from her exploratory questioning and, looking pained, leans in with her palms flat on the table, insisting, ‘but it *is*. The assessment *has to* be more discerning now. We *can't* take everyone in’.

The rare decision to turn a potential service user away once they had made it to the doors of the clinic was now based on the mental health clustering system described in the previous section. The Stepping Stones service had several funding sources but predominantly relied on an NHS contract, which (like Culture in Mind) they had won under the Any Qualified Provider policy (Health and Social Care Act 2012). The contract and the mental health clustering system were based on stipulated upper and lower thresholds of psychological need, which the service was expected to use to define their eligibility criteria. Without workarounds like the one used by K's team, the staff here now had to rethink and refine their client group—separating off those who fell outside of these upper and lower thresholds.

Julie, the administrator at the Stepping Stones service, tells me about a woman who had recently come in for a clinical assessment, having been referred to the service by a primary healthcare professional. Often, I was told, GPs^2^ would refer clients to them, emphasising a trauma or loss they had experienced rather than other needs to justify their eligibility for this service. It had been immediately clear to Julie that something was not right and that even the woman was not sure what she was there for. Only afterwards did Julie discover that this was because the woman had learning difficulties, as well as the mental distress she was experiencing from a recent bereavement. These additional needs had not been recorded on her referral notes, and she had come to the centre alone. Despite seeing the woman's discomfort and confusion, Julie had sent her into a therapy room with one of the clinicians to have her needs and eligibility assessed. In hindsight, she wishes they had not even gone ahead with the assessment because as soon as the woman was in the therapy room, she ‘lost control of her bladder’. Julie and the therapist doing the assessment had helped her get cleaned up and on her way home, but it was clear to them that counselling was not the support this woman needed. She never should have been referred to them; Julie tells me with an apologetic look that ‘she just didn't *belong* here’.

It was true that she probably had been referred to the wrong place and that her needs would not be met with talking therapy alone. But this was as much about an uncontained and out‐of‐place body as it was about falling beyond the upper threshold of need. Because of the additional needs that seemed to have gone unnoticed or uncared for in other services, this woman had become visibly and viscerally out of place in the therapy room. This example stood out because it was a physical manifestation of uncontained need, which in other cases would be about psychological need and therefore less visible and less stark. In interactions like this, I came to know the open‐door policy as a cautionary tale, almost always followed by descriptions of the problems it caused. The director of another centre described the efforts of the Stepping Stones service to maintain an ‘open‐door policy for anyone suffering from loss’ as something that now ‘threatened to sink them’. I was told many times that the increase in people coming to them with additional, pre‐existing and severe mental health needs was a fallout from the cuts to NHS psychiatric services and long‐term health and social care over recent years. It was what had prompted the metaphor of dumping and the threshold work that was deemed necessary to manage this influx of service users.

And so, as well as finding workarounds to allow for ‘overspill’, there was also a need to ‘tidy up’ these breached boundaries and to protect clients already in their care. The opening scene, which introduced the idea of protecting spaces of care from things and people from the outside, highlighted this uncomfortable aspect of the threshold work that care providers were engaged in. Maintaining a safe place for some necessitated the ‘tidying up’ of others. And so, although the material and metaphorical significance of the plea, ‘no dumping!’ grabbed my attention, it does not tell the full story. Its power lies in its expression of feeling—of care providers' sense of being overwhelmed and undervalued in an urban mental healthcare system. What I was observing was the ongoing, if fraught, labour of making and maintaining thresholds of ‘appropriate places’ for ‘appropriate need’.

## 7 Discussion and Conclusions

The aim of this paper has been twofold: first, to take the imperative ‘no dumping!’ as an empirical starting point for understanding how service providers engage in the sociomaterial dynamics of in/exclusion in voluntary sector mental health services; and second, to follow this troubling metaphor, using in‐depth ethnographic observations to disrupt and challenge its immediate associations: of fixed, taken‐for‐granted boundaries between inside and outside, value and waste. To do this, I developed the analytic concept of threshold work. This frames practices of care and inclusion as co‐constituted by both the material limits of place in the city and by upper and lower thresholds of need. It extends conversations in medical sociology on the co‐constituted nature of place and care (Oudshoorn 2011; Bell 2018; Ivanova 2020; Montgomery et al. 2024), attending specifically to the limits of place and care and how they are also shaped by immaterial categories and thresholds of clinical need.

This paper also contributes to the literature which demonstrates the pragmatic, flexible nature of diagnostic thresholds that define what constitutes psychological or psychiatric ‘need’: thresholds that might shift to accommodate the economic logics of improving access to psychological services (Pickersgill 2019) or the microdynamics of mental health practitioners making patients ‘fit’ eligibility criteria (Kienzler 2012). My argument extends this by linking clinical threshold work to sociomaterial and spatial contexts: In this case, the precarious, resource‐scarce and underfunded spaces relied upon to include the excluded. This supports previous ethnographic work by Bister (2017), who observed the sociomaterial shaping of mental healthcare in Berlin. In particular, how ‘classification engages in actions of producing treatability, arranging resources, demarcating responsibilities, [and] practicing accountability’ (Bister 2017, 38). In this paper, the breaching of clinical boundaries—the ‘spilling out’ of existing categories and thresholds of ‘appropriate need’—was shown to be intimately related to concerns about resources, physical and professional capacity, and the growing number of people navigating the mental health system with nowhere else to go for help.

The three interrelated modes of threshold work I have presented become progressively more constrained by the limits of care in their sociomaterial context. ‘Making space’ was arguably the most flexible, with individual care providers deliberately and defiantly breaching the boundaries of the clinic in the face of a literally shrinking clinic space, as the charity sold off therapy rooms and began to deliver care in community settings. ‘Spilling out’ was also a determinedly inclusive mode of threshold work, but it was also fragile and in thrall to more rigid mainstream systems of categorising need and eligibility. The practice of allowing overspill from ‘the IAPT box’ was the result of a painful mismatch between what their NHS contract required of them and their ethical and moral obligations to clients who had ‘nowhere else to go’. The final mode of threshold work, ‘tidying up’, spoke to the uncomfortable relationship between inclusion and exclusion: the making of *people out of place* (Douglas 1966; Ahmed 2000) when they were deemed unsuitable or even unwelcome inside the clinic. I sought to show how this ‘tidying up’ was a result of multiple people and spaces being ‘dumped on’, bearing marks of the failures of welfare, health and social care systems. As community providers are increasingly overburdened, they exclude some to include others and to survive as organisations in the wider landscape of care. Muusse et al. (2020) make the case for understanding deinstitutionalised mental healthcare landscapes in terms of relational, ‘network space’ rather than regional space based on ‘a vision of here and there, each being located on its own side of a boundary’ (2020, 556). The proverbial dump could thus be reframed: characterised not by the ‘waste’ that fills it, but by what the dump is exterior *to* and the relations that define it.

The concept of threshold work has continued relevance in the context of renewed efforts to integrate voluntary and community sector services with statutory mental health under the Community Mental Health Framework (National Collaborating Centre for Mental Health 2019) and local place‐based health policy, intensified by the fallout of the COVID‐19 pandemic (Lorne and Lambert 2025). The aim is to encourage more flexible, community‐based care to reduce the burden on mainstream mental healthcare, but community services continue to be stretched beyond their means to integrate with the mainstream and adapt to new pressures (Sambrook et al. 2025). Despite overall spending increases on mental healthcare since this fieldwork took place, some of the logics of austerity have persisted, particularly around difference and conditionality: ‘the amount of difference allowed to penetrate spaces of the local, to ration its entitlements’ (Gedalof 2018, 23). The modes of threshold work presented in this paper show the voluntary and community sector's responses and resistance to these logics: a rallying around difference and vulnerability under conditions of scarcity and inequitable access. Therefore, tensions between *integration* and *resistance* to mainstream mental healthcare remain stark.

The analysis in this paper offers those designing and implementing integrated community mental healthcare ways to make sense of these tensions in relation to the wider social and material context of living and caring in the city. The need for more flexible pathways and eligibility criteria must be recognised, given recent evidence that current thresholds of need for mental health services are too narrow (Rethink Mental Illness 2025) or too rigid (Sambrook et al. 2025) to provide broad and equitable access to mental healthcare. At the same time, the labour and limits of inclusion must also be made visible. The concept of threshold work helps shift attention away from individualised accounts of exclusion or ‘inappropriate need’ as ad hoc failures of inclusion, towards the wider conditions that make these difficult decisions both necessary and persistent. This includes policies around spending and priorities around who is entitled to what kind of care, but it is also about the politics of space and place: how care is often delivered in un‐cared‐for places, in communities with limited access to other spaces—housing, residential care and safe asylum, to name a few mentioned within this paper.

Threshold work thus emerges in unique and context‐specific ways, but as a concept it can be applied across other spaces of care and care practices, particularly those that are at once inclusive but also limited (by resources, space, values, norms and other social and material modes of organising care). The sociomaterial approach to understanding the making, care and maintenance of thresholds of in/exclusion is one way to disrupt the seemingly simple associations that come with the metaphor of the dumping ground, or indeed any place which is defined by its interiority or exteriority. This speaks to a broader imperative to attend to the particular social, material and spatial conditions that shape problems of in/exclusion in mental healthcare. Rather than problematising the people who are too many or do not fit, we need to problematise the conditions that constrain practices of inclusion. This means paying close critical attention to the never‐finished work of caring for places and who they are for.

## Author Contributions

**Natassia Brenman:** conceptualization, investigation, funding acquisition, writing – original draft, methodology, writing – review and editing, project administration.

## Funding

The research and development of this manuscript was supported by a 3‐year PhD studentship and a 1‐year postdoctoral fellowship [grant reference ES/V011200/1], both awarded by the UKRI Economic and Social Research Council (ESRC).

## Ethics Statement

Ethical approval to conduct this study was obtained from the Ethics Committee of the London School of Hygiene and Tropical Medicine.

## Consent

All staff and service users interviewed and/or formally observed gave written consent in accordance with my ethics protocol.

## Conflicts of Interest

The author declares no conflicts of interest.

## Data Availability

The data that support the findings of this study are available upon request from the corresponding author. The data are not publicly available due to privacy or ethical restrictions.

## Endnotes

^1^
The Mental Health Clustering System is based on an NHS tool developed for care providers to rate the combination and severity of needs, based on their routine screening and assessment process at the point of initial assessment or a moment of change in care. The ‘levels’ of need underpinned how services were commissioned and were used to differing extents in the clinical decision‐making of each service.

^2^
General practitioner, known in other contexts as a family doctor or physician.

## References

[shil70264-bib-0001] Ahmed, S. 2000. Strange Encounters: Embodied Others in Post‐Coloniality. Routledge.

[shil70264-bib-0002] Barad, K. 2003. “Posthumanist Performativity: Toward an Understanding of How Matter Comes to Matter.” Signs: Journal of Women in Culture and Society 28, no. 3: 801–831. 10.1086/345321.

[shil70264-bib-0003] Bell, S. E. 2018. “Placing Care: Embodying Architecture in Hospital Clinics for Immigrant and Refugee Patients.” Sociology of Health & Illness 40, no. 2: 314–326. 10.1111/1467-9566.12604. 29464770

[shil70264-bib-0004] Berlant, L. 2015. “The Commons: Infrastructures for Troubling Times.” Environment and Planning D: Society and Space 34, no. 3: 393–419. 10.1177/0263775816645989.

[shil70264-bib-0005] Bieler, P. , and M. Klausner . 2019. “Niching in Cities Under Pressure. Tracing the Reconfiguration of Community Psychiatric Care and the Housing Market in Berlin.” Geoforum 101: 202–211. 10.1016/j.geoforum.2019.01.018.

[shil70264-bib-0046] Bister, M. D. 2017. “The Concept of Chronicity in Action: Everyday Classification Practices and the Shaping of Mental Health Care.” Sociology of Health & Illness 40, no. 1: 38–52. 10.1111/1467-9566.12623. 28980710

[shil70264-bib-0006] Bister, M. D. , M. Klausner , and J. Niewöhner . 2016. “The Cosmopolitics of ‘Niching’: Rendering the City Habitable Along Infrastructures of Mental Health Care.” In Urban Cosmopolitics : Agencements, Assemblies, Atmospheres, edited by A. Blok and I. Farías , 187–206. Routledge.

[shil70264-bib-0007] Brenman, N. 2023. “At the Edge‐Lands of Mental Healthcare, Whose Vulnerability Counts? With Author’s Commentary on ‘Writing Through Mess’.” In Writing Banal Inequalities: How to Fabricate Stories Which Disrupt, edited by H. Cowan and A. Del Percio , 21–28. Cambridge University Press.

[shil70264-bib-0008] Brenman, N. F. 2019. Place, Need and Precarity in UK Mental Health Care: An Ethnography of Access. PhD thesis. London School of Hygiene and Tropical Medicine.

[shil70264-bib-0009] Brenman, N. F. 2020. “Placing Precarity: Access and Belonging in the Shifting Landscape of UK Mental Health Care.” Culture, Medicine, and Psychiatry [Preprint] 45, no. 1: 22–41. 10.1007/s11013-020-09683-5. PMC7868314 33159271

[shil70264-bib-0010] Busfield, J. 2000. “Introduction: Rethinking the Sociology of Mental Health.” Sociology of Health & Illness 22, no. 5: 543–558. 10.1111/1467-9566.00219.

[shil70264-bib-0011] Cowan, T. L. , and J. Rault . 2022. “Introduction Metaphor as Meaning and Method in Technoculture.” Catalyst: Feminism, Theory, Technoscience 8, no. 2: 1–23. 10.28968/cftt.v8i2.39036.

[shil70264-bib-0012] Cubellis, L. , C. Schmid , and S. von Peter . 2021. “Ethnography in Health Services Research: Oscillation Between Theory and Practice.” Qualitative Health Research 31, no. 11: 2029–2040. 10.1177/10497323211022312. PMC8552374 34286610

[shil70264-bib-0013] De Laet, M. , and A. Mol . 2000. “The Zimbabwe Bush Pump: Mechanics of a Fluid Technology.” Social Studies of Science 30, no. 2: 225–263. 10.1177/030631200030002002.

[shil70264-bib-0014] d’Hoop, A. 2022. “The Moral Life of Doors in an Open Psychiatric Center.” Medical Anthropology 41, no. 1: 67–80. 10.1080/01459740.2021.1992404. 34780310

[shil70264-bib-0015] Douglas, M. 1966. Purity and Danger: An Analysis of Concepts of Pollution and Taboo. Routledge.

[shil70264-bib-0047] Driessen, A. , E. Borgstrom , and S. Cohn . 2021. “Placing Death and Dying: Making Place at the End of Life.” Social Science & Medicine 291: 113974. 10.1016/j.socscimed.2021.113974. 33994221

[shil70264-bib-0016] Fassin, D. 2012. Humanitarian Reason: A Moral History of the Present. University of California Press.

[shil70264-bib-0017] Fitzgerald, D. , R. Ashcroft , G. Hollin , et al. 2020. “Lockdown Texts.” BioSocieties 15, no. 3: 470–499. 10.1057/s41292-020-00199-0.

[shil70264-bib-0018] Foster, L. 2015. In Defence of Welfare. www.social‐policy.org.uk.

[shil70264-bib-0019] Gedalof, I. 2018. Narratives of Difference in an Age of Austerity. 1st ed. 2018 Palgrave Macmillan UK (Thinking Gender in Transnational Times). 10.1057/978-1-137-40065-9.

[shil70264-bib-0020] Gieryn, T. F. 1983. “Boundary‐Work and the Demarcation of Science From Non‐science: Strains and Interests in Professional Ideologies of Scientists.” American Sociological Review 48, no. 6: 781–795. 10.2307/2095325.

[shil70264-bib-0021] Health and Social Care Act . 2012. Health and Social Care Act Chapter 7. http://www.legislation.gov.uk/ukpga/2012/7/contents/enacted.

[shil70264-bib-0022] Ivanova, D. 2020. “Post‐Place Care: Disrupting Place‐Care Ontologies.” Sociology of Health & Illness 42, no. 6: 1296–1311. 10.1111/1467-9566.13100. PMC7384029 32506484

[shil70264-bib-0023] Kasstan, B. 2020. “Haredi (Material) Cultures of Health at the ‘Hard to Reach’ Margins of the State.” In The Material Culture of Failure, edited by T. Carroll , 95–111. Routledge.

[shil70264-bib-0024] Kiely, E. 2021. “Stasis Disguised as Motion: Waiting, Endurance and the Camouflaging of Austerity in Mental Health Services.” Transactions of the Institute of British Geographers 46, no. 3: 717–731. 10.1111/tran.12431.

[shil70264-bib-0025] Kiely, E. 2024. “Between Coercion, Conditionality and Abandonment: A Descriptive Analysis of English Mental Health Spending and Provision Under Austerity.” Journal of Critical Public Health 1, no. 2: 51–73. 10.55016/ojs/jcph.v1i2.78931.

[shil70264-bib-0026] Kienzler, H. 2012. “The Social Life of Psychiatric Practice: Trauma in Postwar Kosova.” Medical Anthropology 31, no. 3: 266–282. 10.1080/01459740.2011.623287. 22540318

[shil70264-bib-0027] Lorne, C. , and M. Lambert . 2025. “Articulating Place: Towards a Conjunctural Analysis of Public Health.” Journal of Critical Public Health [Preprint], 3, no. 1: 123–139. 10.55016/ojs/jcph.vi.79570.

[shil70264-bib-0028] Mattern, S. 2018. “Maintenance and Care.” Places Journal: 1–33. 10.22269/181120.

[shil70264-bib-0029] McGrath, L. , and P. Reavey . 2016. “‘Zip Me Up, and Cool Me Down’: Molar Narratives and Molecular Intensities in ‘Helicopter’ Mental Health Services.” Health & Place 38: 61–69. 10.1016/j.healthplace.2015.12.005. 26798963

[shil70264-bib-0030] Milligan, C. , and D. Conradson . 2006. Landscapes of Voluntarism: New Spaces of Health, Welfare and Governance. Policy Press.

[shil70264-bib-0031] Montgomery, C. M. , A. B. Docherty , S. Humphreys , C. McCulloch , N. Pattison , and S. Sturdy . 2024. “Remaking Critical Care: Place, Body Work and the Materialities of Care in the COVID Intensive Care Unit.” Sociology of Health & Illness 46, no. 3: 361–380. 10.1111/1467-9566.13708. PMC7616248 37702219

[shil70264-bib-0032] Muusse, C. , H. Kroon , C. L. Mulder , and J. Pols . 2020. “Working on and With Relationships: Relational Work and Spatial Understandings of Good Care in Community Mental Healthcare in Trieste.” Culture, Medicine, and Psychiatry 44, no. 4: 544–564. 10.1007/s11013-020-09672-8. PMC7497456 32246246

[shil70264-bib-0033] National Collaborating Centre for Mental Health . 2019. The Community Mental Health Framework for Adults and Older Adults: Support, Care and Treatment.

[shil70264-bib-0034] Oudshoorn, N. 2011. “How Places Matter: Telecare Technologies and the Changing Spatial Dimensions of Healthcare.” Social Studies of Science 42, no. 1: 121–142. 10.1177/0306312711431817. 22530385

[shil70264-bib-0035] Parr, H. 1997. “Mental Health, Public Space, and the City: Questions of Individual and Collective Access.” Environment and Planning D: Society and Space 15, no. 4: 435–454. 10.1068/d150435.

[shil70264-bib-0036] Parr, H. 2000. “Interpreting the ‘Hidden Social Geographies’ of Mental Health: Ethnographies of Inclusion and Exclusion in Semi‐ Institutional Places.” Health & Place 6, no. 3: 225–237. 10.1016/s1353-8292(00)00025-3. 10936777

[shil70264-bib-0037] Pickersgill, M. 2019. “Access, Accountability, and the Proliferation of Psychological Therapy: On the Introduction of the IAPT Initiative and the Transformation of Mental Healthcare.” Social Studies of Science 49, no. 4: 627–650. 10.1177/0306312719834070. PMC7613166 30905265

[shil70264-bib-0038] Rethink Mental Illness . 2025. Right Treatment, Right Time 2025: The True Cost of Mental Health Waiting Times. Rethink Mental Illness. https://www.rethink.org/campaigns‐and‐policy/campaign‐with‐us/resources‐and‐reports/right‐treatment‐right‐time‐2025‐the‐true‐cost‐of‐mental‐health‐waiting‐times/.

[shil70264-bib-0039] Rose, N. , and D. Fitzgerald . 2022. The Urban Brain: Mental Health in the Vital City. Princeton University Press.

[shil70264-bib-0040] Sambrook, L. , J. C. McIntyre , R. Nathan , et al. 2025. “‘A Community in Crisis’: Staff Qualitative Experiences of NHS and Third Sector Mental Healthcare in England.” BJPsych Open 11, no. 5: e210. 10.1192/bjo.2025.10826. PMC12451547 40936463

[shil70264-bib-0041] Shevlin, M. , S. Butter , O. McBride , et al. 2021. “Refuting the Myth of a ‘Tsunami’ of Mental Ill‐Health in Populations Affected by COVID‐19: Evidence That Response to the Pandemic Is Heterogeneous, Not Homogeneous.” Psychological Medicine: 1–9. 10.1017/S0033291721001665. PMC8111207 33875044

[shil70264-bib-0042] Traill, H. , S. Anderson , D. Shaw , A. Cumbers , and R. McMaster . 2024. “Caring at the Edges: Infrastructures of Care and Repair in Urban Deprivation.” Environment and Planning D: Society and Space 42, no. 2: 190–210. 10.1177/02637758241231106.

[shil70264-bib-0043] Vertovec, S. 2007. “Super‐Diversity and Its Implications.” Ethnic and Racial Studies 30, no. 6: 1024–1054. 10.1080/01419870701599465.

[shil70264-bib-0044] Wright, F. 2022. “Making Good of Crisis: Temporalities of Care in UK Mental Health Services.” Medical Anthropology 41, no. 3: 315–328. 10.1080/01459740.2021.2018586. 35060803

